# Characteristics and Pattern of Calcified Nodule and/or Nodular Calcification Detected by Intravascular Ultrasound on the Device-Oriented Composite Endpoint (DoCE) in Patients with Heavily Calcified Lesions Who Underwent Rotational Atherectomy-Assisted Percutaneous Coronary Intervention

**DOI:** 10.1155/2023/6456695

**Published:** 2023-01-21

**Authors:** Ploy Pengchata, Rungtiwa Pongakasira, Namthip Wongsawangkit, Asa Phichaphop, Nattawut Wongpraparut

**Affiliations:** ^1^Division of Cardiology, Department of Medicine, Faculty of Medicine Siriraj Hospital, Mahidol University, Bangkok, Thailand; ^2^Her Majesty's Cardiac Center, Faculty of Medicine Siriraj Hospital, Mahidol University, Bangkok, Thailand

## Abstract

**Objectives:**

This study aimed to determine characteristics and pattern of a calcified nodule (CN) and/or nodular calcification (NC) detected by intravascular ultrasound (IVUS) on the device-oriented composite endpoint (DoCE) in patients with calcified lesions who underwent rotational atherectomy (RA)-assisted percutaneous coronary intervention (PCI).

**Background:**

The characteristics and pattern of a CN and/or NC on clinical outcome remain unknown.

**Methods:**

We retrospectively enrolled patients who underwent RA-assisted PCI at Siriraj Hospital during August 2016 to April 2020. Preprocedural IVUS imaging was mandatory. CN/NC was defined as convex shape of luminal surface and luminal side of calcium with protrusion into the coronary artery lumen as assessed by IVUS. The primary outcome was cumulative of DoCE, defined as the composite of cardiovascular death, myocardial infarction, and clinically-driven target lesion revascularization.

**Results:**

Two hundred patients were included. Primary outcome occurred in 14%. The cumulative DoCE was significantly higher in the CN/NC group than that in the non-CN/NC group (20.7% vs. 8.8%, *p* = 0.022). CN/NC (*p* = 0.023) and MSA ≤ 5.5 mm^2^ (*p* = 0.047) were correlated with a significantly higher cumulative DoCE. CN/NC was the independent predictor for the cumulative DoCE (HR = 2.96, 95% CI 1.08–8.11, *p* = 0.035). Pattern and characteristic of CN/NC have a prognostic value. Patients with an eccentric CN/NC had a significantly higher cumulative DoCE compared to those CN/NC with concentric calcification (*p* = 0.014).

**Conclusion:**

The presence of a CN/NC in patients with heavily calcified lesions who underwent RA-assisted PCI was found to be associated with increased cumulative 5 year DoCE, especially in patients with an eccentric CN/NC. The clinical trial is registered with TCTR20210616001.

## 1. Introduction

A calcified nodule (CN) is defined as an eruptive nodular calcification that protrudes into the coronary artery lumen. Eruptive CN is the cause of acute coronary syndrome (ACS) in about 2–8% of ACS patients [1]. The outcomes of patients with a CN in native coronary artery disease (CAD) were first reported by Xu et al. [2]. Nodular calcification (NC) is benign in nature that does not lead to coronary thrombosis, but it could limit the device cross ability, stent strut damage, stent malapposition, and stent under expansion. To differentiate between these 2 pathological entities is very difficult. In the setting of acute coronary syndrome (ACS), the appearance of CN at the culprit lesion is suggestive of eruptive CN. Nonculprit CN was observed in 30% of ACS. Morofuji et al. found a 41% prevalence of CN in patients with heavily calcified lesion that required rotational atherectomy (RA)-assisted percutaneous coronary intervention (PCI) [3]. They also reported CN to be associated with an unfavorable post-PCI outcome and significantly increased incidence of 5 year major adverse cardiovascular event (MACE). However, they did not identify the characteristics of CN/NC that associate with adverse clinical outcome. Plaque modification is an important step when managing a heavily calcified lesion, and plaque modification in a CN/NC can be a challenge. Intravascular ultrasound (IVUS) has a significant role in assisted coronary and noncoronary intervention [4–12]. Intravascular imaging guidance reduce 1 year MACE in the patients with heavy calcified lesion undergoing RA-assisted PCI [13]. Information obtained from intravascular ultrasound (IVUS) specific to the presence and characteristics of a CN/NC within a heavily calcified lesion that impact on device-oriented composite endpoint (DoCE) will improve patient management and clinical outcomes.

The aim of this study was to determine the impact of a CN/NC detected by IVUS on the DoCE in patients with heavily calcified lesions who underwent RA-assisted PCI. Our secondary objective was to determine the characteristics of a CN/NC identified by IVUS that associate with unfavorable clinical outcomes.

## 2. Materials and Methods

### 2.1. Study Design

This single-center retrospective cohort study was conducted at the Interventional Cardiology Unit of the Division of Cardiology, Department of Medicine, Faculty of Medicine Siriraj Hospital, Mahidol University, Bangkok, Thailand. Two hundred and ninety-three consecutive patients who underwent RA-assisted IVUS-guided PCI to treat a moderate to severe calcified lesion during August 2016 to April 2020 were evaluated. Preprocedural IVUS was mandatory, and preprocedural IVUS imaging could have been performed either before or after RA. Patients with significant valvular heart disease or severe comorbidities, such as malignant neoplasm requiring chemotherapy, surgery, or radiation, were excluded. We also excluded patients with cardiogenic shock before procedure, and patients who were lost to follow-up. Two hundred patients who met inclusion and exclusion criteria were enrolled. The Siriraj Institutional Review Board approved this study (COA no. 220/2021).

Histological section classification of calcified nodule was described by Virmani et al. [14, 15]. Calcified nodule (CN) is defined as a lesion with fibrous cap disruption from eruptive calcific nodules associated with an occlusive or nonocclusive platelet/fibrin thrombus. Nodular calcification (NC) is the least common form of calcification in the coronary vasculature. NC is accompanied by fibrin with a thick, intact fibrous cap. In patients with heavily calcified lesions undergoing RA, the advancement of the IVUS catheter before intervention is impossible. The resolution of IVUS in a detected fibrous cap disruption is limited, especially in IVUS with lower MHz. The study objective is focused on impact of these CN/NC on DoCE in patients with heavily calcified lesions who underwent RA-assisted PCI. We opt to combine these two entities for analysis.

CN/NC was defined as convex shape of luminal surface and luminal side of calcium with protrusion into the coronary artery lumen [1, 16] as assessed by IVUS. Patients were categorized into either the CN/NC group (presence of at least one CN/NC within the target lesion) or the non-CN/NC group.

### 2.2. Procedures

#### 2.2.1. IVUS Analysis and Identification of CN/NC

All study patients had prestent IVUS imaging. IVUS-guided RA using either the OptiCross™ (Frequency 60 MHz, Boston Scientific, Marlborough, MA, USA) or Eagle Eye Platinum™ (Frequency 20 MHz, Philips, Rancho Cordova, CA, USA) system was used depending on the decision of the operator. OptiCross™ was used in 45% of patients in this study. Thirty-six (20%) patients IVUS was obtained prior RA. One hundred and sixty-four patients (80%) the IVUS catheter could not pass the lesion before RA. IVUS imaging obtained after RA.

An experienced intravascular imaging technician and experienced interventionists who were blinded to the clinical outcome reviewed the angiographic and IVUS imaging data. Intraobserver and interobserver variability yielded good concordance for the diagnosis of CN (*k* = 0.95 and *k* = 0.90, respectively).

#### 2.2.2. Angiographic Analysis

Qualitative and quantitative analysis was performed by an experienced intravascular imaging technician and 2 experienced interventional cardiologists who were blinded to the clinical information. Quantitative coronary angiography (QCA) analysis was performed using a cardiovascular measurement system (QAngio XA 7.2, MEDIS, Leiden, The Netherlands). Coronary angiograms were performed in at least 2 orthogonal views. Angiographic calcification grading was as follows: (1) none: no radiopacity; (2) mild calcification: faint radiopacities noted during the cardiac cycles; (3) moderate calcification: dense radiopacities noted only during the cardiac cycle; and (4) severe calcification: dense radiopacities noted without cardiac motion before contrast injection generally compromising both sides of the arterial lumen.

#### 2.2.3. IVUS Quantitative Analysis

Qualitative and quantitative analysis was performed at the target lesion according to and following an expert consensus document on the standards for acquisition, measurement, and reporting of intravascular ultrasound studies [17].

(1) Maximum calcium arch grade at the lesion is as follows:
  - Eccentric calcification was defined as a calcium arch <180 degrees
  - Concentric calcification was defined as a calcium arch ≥180 degrees

(2) Calcium location is as follows:
  - Superficial calcification was defined as the leading edge of the acoustic shadowing appears within the shallowest 50% of the plaque plus media thickness
  - Deep calcification was defined as the leading edge of the acoustic shadowing appears within the deepest 50% of the plaque plus media thickness

(3) Calcium characteristics are as follows:
  - Napkin ring calcification was defined as severe circumferential calcification.
  - Eccentric calcified nodule/nodular calcification was defined as calcified nodule/nodular calcification without calcification at the opposite site of calcified nodule/nodular calcification.
  - Calcified nodule/nodular calcification with concentric calcification was defined as calcified nodule/nodular calcification with calcification at the opposite site of calcified nodule/nodular calcification. Mix lesion was identified as concentric calcification.
  - Reverberation was defined as an artifact represented by secondary false echoes of the same structure and reverberation from the leading edge of calcium [18].
  - Calcium fracture was defined as a gap of calcium and direct exposure of calcium to the lumen at the gap [19]. Lumen measurements were performed using the interface between the lumen and the leading edge of the intima.
  - Lumen cross-sectional area (CSA) was defined as the area bounded by the luminal border.

(4) Reference vessel is as follows:
  - The proximal and distal reference segments with the maximum lumen and least amount of plaque within 5 mm proximal or distal to the lesion

(4) Stent measurement is as follows:
  - Minimal stent area (MSA) was defined as the minimal area bounded by the stent border
  - Minimum stent diameter was defined as the shortest diameter through the center of mass of the stent
  - Maximum stent diameter was defined as the longest diameter through the center of mass of the stent
  - Asymmetry index [20] was defined as follows: ((maximum stent diameter minus minimum stent diameter) divided by maximum stent diameter) measured at the MSA
  - Asymmetrical stent expansion was defined as a stent symmetry index >0.3
  - Symmetrical stent expansion was defined as a stent symmetry index ≤0.3

### 2.3. PCI Procedure and Clinical Follow-Up

All patients received a bolus injection of heparin 100 unit/kilogram to maintain an activated clotting time of >250 seconds. Dual antiplatelet therapy with 81 mg/day aspirin with 75 mg/day clopidogrel or 10 mg/day prasugrel or 90 mg twice a day ticagrelor was continued for at least one year after the procedure. The conventional 0.014 inch guidewire was replaced with a 0.009 inch ROTAWire™ floppy guidewire (Boston Scientific) or a 0.009 inch ROTAWire™ extra support guidewire (Boston Scientific). The initial burr size selection was based on preprocedural IVUS imaging indicating whether or not the lesion can be passed before RA. A burr speed of 140,000–200,000 revolutions per minute (RPM) with a run duration of 10–15 seconds was used. The target final burr-to-artery ratio was within 0.4 to 0.6. Noncompliance balloon or cutting balloon was routinely used for predilatation before stent implantation. The stent diameter was determined by measuring the external elastic lamina diameter at the proximal and distal reference sites. Postprocedure imaging was performed after stent implantation to evaluate stent apposition, stent optimization, and procedural complication, such as dissection or tissue protrusion. Duration and type of antiplatelet and antithrombotic were prescribed according to operator discretion.

### 2.4. Study Outcome

The primary outcome was the device-oriented composite endpoint (DoCE), defined as the composite of cardiovascular death, myocardial infarction, and clinically-driven target lesion revascularization.

### 2.5. Statistical Analysis

The sample size was calculated using data from a study by Morofuji, et al. [3]. They reported an event rate of 24% in the CN group, and an 8.3% event rate in the non-CN group. We estimated that a sample size of 170 patients would give our study 80% power to detect a 15% difference in DoCE between the CN/NC group and non-CN/NC group. Continuous variables were reported as median (interquartile range 25–75) or mean ± standard deviation depending on the distribution of data. Categorical data were reported as number and percentage. Chi-square test or Fisher's exact test was used to compare categorical data, and Student's *t*-test (normally distributed data) or Mann–Whitney *U* test (non-normally distributed data) was used to compare continuous data. Kaplan-Meier survival analysis was used to estimate the cumulative DoCE and Cox proportional hazard ratio was used to identify independent predictors of the cumulative DoCE. Factors with *p* value <0.2 after using a univariate model were included in multivariable Cox proportional hazard analysis. A *p* value less than 0.05 was considered to be statistically significant. All statistical analyses were performed using SPSS Statistics version 18 (SPSS, Inc., Chicago, IL, USA).

## 3. Results

**Figure 1:**
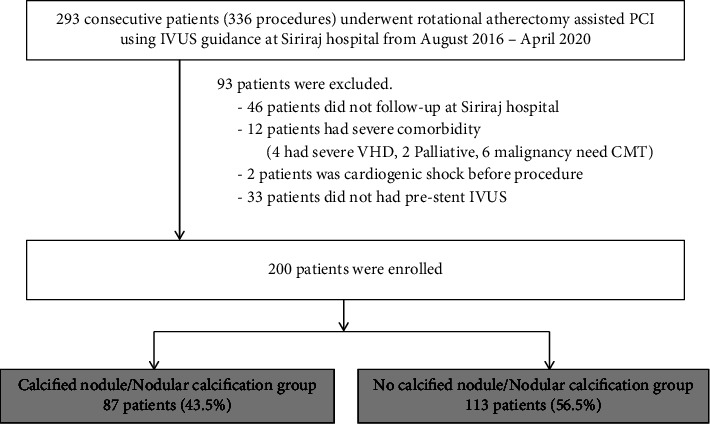
Flow diagram describing the patient enrollment protocol. PCI: percutaneous coronary intervention and IVUS: intravascular ultrasound.

The study reviewed 293 consecutive patients who underwent RA-assisted IVUS-guided PCI at the Faculty of Medicine, Siriraj Hospital, during August 2016 to April 2020. Two hundred patients who met inclusion and exclusion criteria were enrolled. A flow diagram showing the patient enrollment protocol is shown in Figure 1.

### 3.1. Baseline Characteristics

The prevalence of history of hypertension was significantly higher in the CN/NC group than in the non-CN/NC group. No other baseline characteristics were significantly different between groups. Forty-three percent of overall patients had chronic kidney disease. The baseline characteristics of all patients and comparison between CN/NC and non-CN/NC patients are shown in Table 1.

### 3.2. Lesion and Procedure Characteristics

Seventy-seven percent of overall patients had angiographic severe calcification. The majority of patients had multivessel disease, and 71% of procedures were performed in the left anterior descending (LAD) artery. Lesion and procedural characteristics are summarized in Table 2. Final burr size, burr-to-artery ratio, stent diameter, number of stents, and stent length were not significantly different between groups. Periprocedural complications (Table 2) occurred in 3.0% of overall patients, and they occurred more frequently in the CN group (4.6% *vs.* 1.8%, *p* = 0.407).

IVUS analysis showed superficial calcification and napkin ring calcification in 98% and 50% of overall cases, respectively. CN/NC was found in 43.5% (87 patients) of overall patients. Regarding calcification characteristics, superficial calcification, concentric calcification, napkin ring calcification, and calcium fracture were not significantly different between the CN/NC and non-CN/NC groups. For lesion subtypes, concentric calcification without CN/NC were found most frequently in 102 patients (51%) followed by CN/NC with concentric calcification (80 patients, 40%). Eccentric calcification was found in 18 patients (9%). Eccentric CN/NC were the least common subtypes of calcified plaque in this study (7 patients, 3.5%). Poststent IVUS analysis showed no significant difference in mean MSA between groups. Asymmetrical stent expansion was more frequently observed (numerically) in the CN/NC group (9.3% *vs*. 1.4%, *p* = 0.084) (Table 3).

### 3.3. Clinical Outcomes

**Figure 2:**
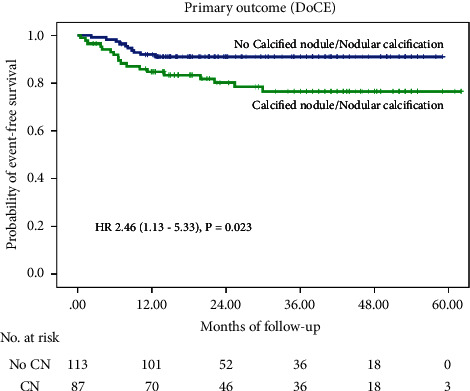
Kaplan-Meier survival analysis for the primary outcome after RA-assisted intravascular ultrasound (IVUS)-guided PCI compared between those with and without calcified nodule/nodular calcification. DoCE: device-oriented composite endpoint; RA: rotational atherectomy; IVUS: intravascular ultrasound; and PCI: percutaneous coronary intervention.

**Figure 3:**
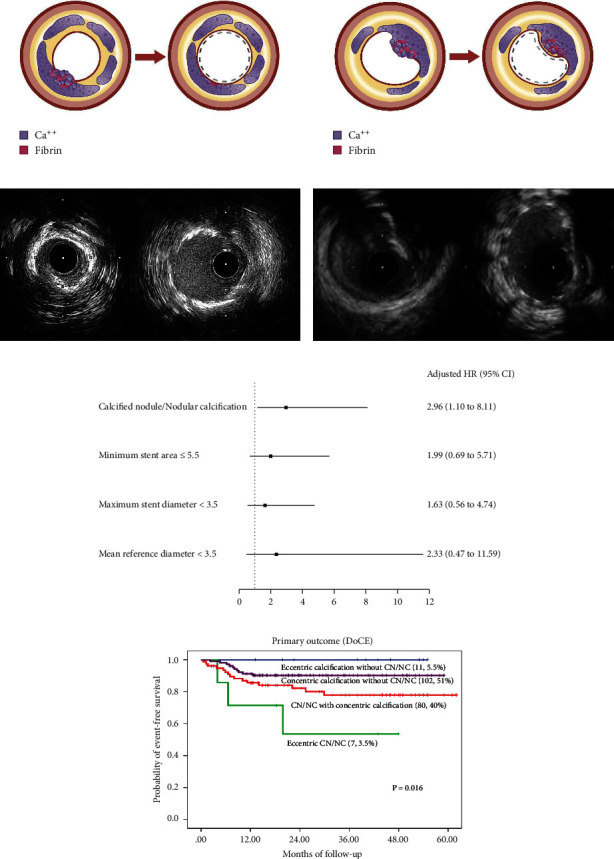
Central illustration. Drawing and IVUS results pre- and post-stent implantation, comparison of primary outcome according to calcific plaque and characteristic and pattern of calcified nodule/nodular calcification are presented. (a) Drawing and IVUS imaging showed pre- and post-stent for concentric calcification with calcified nodule/nodular calcification. (b) Drawing and IVUS imaging showed pre- and post-stent for an eccentric calcified nodule/nodular calcification. (c) A forest plot showing the adjusted hazard ratios and 95% confidence intervals for the primary outcome. (d) Kaplan-Meier survival analysis for the primary outcome after RA-assisted intravascular ultrasound (IVUS)-guided PCI compared among different subtypes of calcific plaque and calcified nodule/nodular calcification characteristics. IVUS: intravascular ultrasound; DoCE: device-oriented composite endpoint; RA: rotational atherectomy; PCI: percutaneous coronary intervention; CN: calcified nodule; and NC: nodular calcification.

The longest follow-up was 62 months, with a median follow-up duration of 26 months (interquartile range [IQR]: 14, 44 months). The primary outcome occurred in 14% (28 patients), CV death occurred in 7% (14 patients), MI occurred in 5% (10 patients), there was no stent thrombosis, and CDTLR occurred in 4.5% (9 patients) of overall patients. Incidence of the primary outcome was significantly higher in the CN/NC group (20.7% vs. 8.8%, *p* = 0.023, hazard ratio (HR): 2.46). Incidence of CV death was higher in the CN/NC group (9.2% vs. 5.3%, *p* = 0.296, hazard ratio (HR): 1.76). Incidence of MI was higher in the CN/NC group (6.9% vs. 3.5%, *p* = 0.277, hazard ratio (HR): 2.02). Incidence of CDTLR was higher in the CN/NC group (6.9% vs. 2.7%, *p* = 0.168, hazard ratio (HR): 2.65). Kaplan-Meier survival analysis estimating the cumulative incidence of the DoCE compared between the CN/NC and non-CN/NC groups is shown in Figure 2. After univariate analysis, we found that the cumulative DoCE was significantly higher in the CN/NC (unadjusted HR 2.46, 95% CI 1.13–5.33, *p* = 0.023), MSA ≤ 5.5 mm^2^ (unadjusted HR 2.57, 95% CI 1.01–6.53, *p* = 0.047). After multivariate analysis, CN/NC was found to be independent predictor for the cumulative DoCE (adjusted HR 2.96, 95% CI 1.10–8.11, *p* = 0.035). Significant difference in the cumulative DoCE was observed among the 4 calcified lesion phenotype characteristic groups (Figure 3, central illustration).

Wire bias based on IVUS data and DoCE in the patients with eccentric CN/NC is shown in Table 4. The patient who had wire bias toward eccentric CN/NC had a good prognosis after RA-assisted PCI. In contrast, the patient who had wire bias opposite to eccentric CN/NC had increased 5 year DoCE. Sixty percent of the patients with eccentric CN/NC and wire bias opposite to CN/NC had 5 year DoCE.

## 4. Discussion

The present study investigated the impact of a CN/NC on the DoCE in patients with a heavily calcified lesion who underwent RA-assisted IVUS-guided PCI. The main findings of the study are as follows: (1) a CN/NC was found in 43.5% of overall patients; (2) the presence of a CN/NC significantly increased the likelihood of occurrence of the DoCE within 5 years after PCI compared to non-CN/NC patients; (3) CN/NC and MSA ≤ 5.5 mm^2^ were correlated with significantly higher cumulative DoCE, but only CN/NC was an independent predictor for the cumulative DoCE; (4) the characteristic and pattern of CN/NC detected by IVUS have the prognostic value in the predicted long-term DoCE. The CN/NC with concentric calcification has acceptable long-term DoCE to calcified lesion without CN/NC. The eccentric CN/NC had the worst cumulative 5 year DoCE; and (5) the wire bias opposite to eccentric CN/NC based on IVUS data had increased 5 year DoCE.

The 43% prevalence of CN/NC observed in this study is consistent with the 48% rate reported by Morofuji et al. [3]. Compared to their study, our study had more patients with ACS (24.1% vs. 12.9%) and less patients on hemodialysis (10.6% vs. 29.2%). The mean RVD and mean MSA were both larger in our study (3.19 vs. 2.47 mm and 6.38 vs. 4.67 mm^2^, respectively). The 5 year DoCE was similar between the studies (14% vs. 16.4%). Our study found DoCE to be driven by TVR, whereas DoCE was driven by TVR and stent thrombosis in the Morofuji et al. study. This difference between groups may be explained by less hemodialysis, larger vessel size, and more use of third-generation thienopyridine and ticagrelor.

Prestent and Poststent IVUS analysis helps in risk prediction in PCI. Zhang M et al. reported IVUS derived calcium score using (1) superficial calcium angle >270° longer than 5 mm, (2) 360° of superficial calcium, (3) calcified nodule, and (4) vessel diameter <3.5 mm [21]. These parameters of IVUS derived calcium score associated with stent underexpansion and requiring plaque modification such as RA before stent implantation. Even though napkin ring calcification is a predictor of stent under expansion [22] and an indication for plaque modification, such as RA, napkin ring calcification or superficial calcium >270° longer than 5 mm has no impact on the long-term DoCE in our study because we performed RA in all patients. In the setting of RA-assisted PCI, we found the calcified nodule and MSA < 5.5 mm^2^ correlated with a significantly higher incidence of the cumulative DoCE. MSA ≤ 5.5 mm^2^ is also the cut point for predicting angiographic in-stent restenosis in the DES era [23]. However, after multivariate analysis, the CN/NC was an independent predictor for the cumulative DoCE.

In our study, the prevalence of MSA ≤ 5.5 mm^2^ and stent expansion were not significantly different between the CN/NC and non-CN/NC groups, but TVR was significantly higher in the CN/NC group [24]. CN also has known to increase the incidence of TVR. Nakamura et al. proposed the potential mechanism of progression of in-stent calcified nodule to be a protrusion of calcified nodule followed by calcifying fibrin thrombus in the early phase, fragments of calcification and fibrin deposition caused by mechanical destruction of sheath of calcification in mid-phase, and neocalcified nodule in the late phase [25]. Asymmetrical stent expansion was also numerically higher in the CN/NC group. Suwannasom et al. [20] found asymmetrical stent expansion to be the independent factor associated with device-oriented composite endpoint (DoCE).

In the present study, we used pre- and post-PCI IVUS analysis to investigate pattern and characteristic of a CN/NC in predicting the cumulative DoCE. The pattern and distribution of a CN/NC in relation to surrounding calcified lesion obtained by prestent IVUS analysis helps in risk prediction. Pre-stent IVUS analysis showed an eccentric CN/NC to be associated with a higher cumulative DoCE compared to a CN/NC with concentric calcification. The presence of concentric calcification or napkin ring calcification with a CN/NC would allow the CN/NC to be adequately ablated during RA. In contrast to an eccentric CN/NC, RA would ablate in the opposite side of CN/NC particularly in the presence of vessel tortuosity or wire bias. This mechanism could affect suboptimal contact of RA burr to the CN/NC leading to inadequately plaque modification/lesion preparation specifically in an eccentric CN/NC lesion. Based in IVUS data in this study, we found that the patient with eccentric CN/NC and wire bias toward CN/NC still had favorable prognosis after RA-assisted PCI. The patient with eccentric CN/NC and wire bias opposite to CN/NC had increased 5 year DoCE. The character of wire bias opposite to CN/NC in eccentric CN/NC was probable unsuitable anatomy for RA-assisted PCI. We suggested paying attention on wire bias characteristics based on IVUS data in the patients with eccentric CN/NC in order to select appropriate atherectomy device during the procedure. Stent implantation in an eccentric CN/NC will preferentially limit expansion on calcified side and overexpansion on the opposite noncalcified site causing stent malapposition/asymmetrical stent expansion. Using of the high-pressure noncompliance or semicompliance balloon postdilatation may increase risk for vessel perforation on noncalcified site [26]. Watanabe et al. [27] found that stent malapposition was more frequently observed in calcified nodule. Compared to PCI without RA, RA did not reduce the risk of stent malapposition or MACE in Watanabe study. An eccentric CN/NC was presented in 44% in Watanabe study that could explain a higher DoCE in RA-assisted PCI.

Our study provides novel insights on the prognostic value of different coronary plaque calcification phenotypes. Our findings suggest that careful attention should be paid to both pre-PCI and poststent IVUS analysis. When an eccentric CN/NC is encountered, we recommend careful attention on the effect of wire bias and/or selection of the proper rota-wire type to facilitate optimal burr contact with the CN/NC. Periprocedural IVUS assessment for adequate plaque modification, lesion preparation before stent implantation is crucial to reduce the possibility of stent under expansion, asymmetrical expansion, and incomplete stent apposition. Other debulking techniques, such as orbital atherectomy and intravascular lithotripsy, should also be investigated in this subtype of lesion (CN/NC with eccentric calcification).

### 4.1. Limitations

This study has some mentionable limitations. First, the retrospective design of our study increased its vulnerability to missing or incomplete data. Second, our data was collected from only a single center. Third, our center is a major urban national tertiary referral center for the complex PCI. As such, it is possible that our findings may not be generalizable to other care settings. Fourth, we used routine RA as debulking therapy in calcified lesion. We have no outcome information of other debulking therapy such as orbital atherectomy or intravascular lithotripsy on CN. Fifth, patients' data were analyzed from RA database. We cannot exclude selection bias in imaging characteristics which is suitable for RA. Eccentric CN/NC was found in the minority group in our study while it was found in the majority group in Watanabe study [27]. Sixth, because of the heavy calcified lesion with significant stenosis, 80% of patients IVUS failed to cross lesion prior to RA. However, IVUS was immediately performed after RA, prior to angioplasty in all patients. Even though, the characteristic and pattern of calcified nodule may be able to interpret. But we could miss the important information such as disrupt or intact fibrous cap, eruptive calcified nodule from morphological change post RA. Seventh, IVUS has limited resolution. IVUS could miss to detect fibrous cap disruption and the presence of luminal thrombus on the surface of CN; hence, it cannot clearly differentiate CN from NC. Optical coherence tomography (OCT) would provide a better differentiation between CN and NC. However, this study focused on impact of CN/NC on the DoCE rather than ACS etiology.

## 5. Conclusions

The presence of a calcified nodule or nodular calcification in patients with heavily calcified lesions who underwent RA-assisted PCI was found to be associated with increased cumulative DoCE, especially in patients with an eccentric calcified nodule or nodular calcification and wire bias opposite to the calcified nodule.

## Figures and Tables

**Table 1:** Baseline characteristics of all patients and comparison between those with and without calcified nodule.

Characteristics	All patients (*N* = 200)	CN (*n* = 87)	Non-CN (*n* = 113)	*p*
Age (years)	72.5 ± 9.4	72.7 ± 10.1	72.4 ± 8.8	0.832
Male gender	106 (53.0%)	48 (55.2%)	58 (51.3%)	0.589
Diabetes mellitus	113 (56.5%)	53 (60.9%)	60 (53.1%)	0.269
Hypertension	193 (96.5%)	87 (100.0%)	106 (93.8%)	0.019
Dyslipidemia	150 (75.0%)	64 (73.6%)	86 (76.1%)	0.681
Chronic kidney disease	87 (43.5%)	36 (41.4%)	51 (45.1%)	0.596
Hemodialysis	20 (10.0%)	6 (6.9%)	14 (12.4%)	0.199
Prior MI	43 (21.5%)	17 (19.5%)	26 (23.0%)	0.554
Prior PCI	17 (8.5%)	7 (8.0%)	10 (8.8%)	0.840
Prior CABG	16 (8.0%)	7 (8.0%)	9 (8.0%)	0.983
Current smoker	9 (4.5%)	5 (5.7%)	4 (3.5%)	0.506
LVEF (%)	61 (46, 69)	62 (47, 70)	61 (46, 67)	0.592
LVEF <30%	8 (4.3%)	3 (3.7%)	5 (4.7%)	1.000
Atrial fibrillation	25 (12.5%)	10 (11.5%)	15 (13.3%)	0.706
Clinical presentation				
CCS	154 (77.0%)	64 (73.6%)	90 (79.6%)	0.311
ACS	46 (23.0%)	23 (26.4%)	23 (20.4%)	
Medication at discharge				
Aspirin	199 (99.5%)	87 (100.0%)	112 (99.1%)	1.000
P2Y12 inhibitors				
Clopidogrel	166 (83.0%)	74 (85.1%)	92 (81.4%)	
Ticagrelor	29 (14.5%)	11 (12.6%)	18 (15.9%)	0.563
Prasugrel	4 (2.0%)	1 (1.1%)	3 (2.7%)	
Oral anticoagulant	19 (9.5%)	7 (8.0%)	12 (10.6%)	0.538
Statin	195 (97.5%)	84 (96.6%)	111 (98.2%)	0.655

Data presented as frequency and percentage, mean ± standard deviation, or median and interquartile range. A *p* value<0.05 indicates statistical significance. CN, calcified nodule; MI, myocardial infarction; PCI, percutaneous coronary intervention; CABG, coronary artery bypass graft; LVEF, left ventricular ejection fraction; CCS, chronic coronary syndrome; and ACS, acute coronary syndrome.

**Table 2:** Lesion and procedure characteristics of all patients and comparison between those with and without calcified nodule.

Characteristics	All patients (*N* = 200)	CN (*n* = 87)	Non-CN (*n* = 113)	*p*
Target vessel				
Left main	8 (4.0%)	4 (4.6%)	4 (3.5%)	
Left anterior descending	145 (72.5%)	60 (69.0%)	85 (75.2%)	0.606
Left circumflex	8 (4.0%)	5 (5.7%)	3 (2.7%)	
Right coronary artery	39 (19.5%)	18 (20.7%)	21 (18.6%)	
Number of vessel disease				
1	45 (22.5%)	16 (18.4%)	29 (25.7%)	
2	78 (39.0%)	34 (39.1%)	44 (38.9%)	0.602
3	68 (34.0%)	33 (37.9%)	35 (31.0%)	
Left main	9 (4.5%)	4 (4.6%)	5 (4.4%)	
Lesion location				
Ostial	11 (5.5%)	7 (8.0%)	4 (3.5%)	0.215
Proximal	121 (60.5%)	50 (57.5%)	71 (62.8%)	0.442
Mid	105 (52.5%)	43 (49.4%)	62 (54.9%)	0.445
Distal	12 (6.0%)	6 (6.9%)	6 (5.3%)	0.639
Angiographic moderate to severe calcification	195 (97.5%)	86 (98.9%)	109 (96.5%)	0.390
Moderate	40 (20.0%)	17 (19.5%)	23 (20.4%)	0.583
Severe	155 (77.5%)	69 (79.3%)	86 (76.1%)	
Chronic total occlusion lesion	10 (5.0%)	3 (3.4%)	7 (6.2%)	0.518
Ostial lesion	61 (30.5%)	28 (32.2%)	33 (29.2%)	0.650
Bifurcation	64 (32.0%)	28 (32.2%)	36 (31.9%)	0.961
Stent type				
Bare metal stent	5 (2.5%)	3 (3.5%)	2 (1.8%)	0.654
Drug-eluting stent	193 (97.5%)	83 (96.5%)	110 (98.2%)	
Number of stents				
1	96 (48.5%)	42 (48.8%)	54 (48.2%)	0.931
>1	102 (51.5%)	44 (51.2%)	58 (51.8%)	
Max stent diameter	3.0 (3.0, 3.5)	3.5 (3.0, 3.5)	3.0 (2.8, 3.5)	0.292
Stent length	38.0 (28.0, 52.0)	36.5 (28.0, 52.0)	38.0 (29.0, 52.0)	0.733
Cutting or scoring balloon	17 (8.5%)	5 (5.7%)	12 (10.6%)	0.221
Max balloon	3.3 (3.0, 3.5)	3.0 (2.8, 3.6)	3.3 (3.0, 3.5)	0.970
Rotational atherectomy				
Final burr	1.5 (1.5, 1.8)	1.5 (1.5, 1.8)	1.5 (1.5, 1.8)	0.217
Burr-to-artery ratio	0.49 ± 0.09	0.49 ± 0.10	0.49 ± 0.09	0.740
>1 burr	47 (23.5%)	19 (21.8%)	28 (24.8%)	0.627
Perioperative complications	10 (5.0%)	8 (9.2%)	2 (1.8%)	0.022
Dissection and acute closure	4 (2.0%)	2 (2.3%)	2 (1.8%)	1.000
Perforation	0 (0.0%)	0 (0.0%)	0 (0.0%)	—
Slow flow or IABP	2 (1.0%)	2 (2.3%)	0 (0.0%)	0.188
Myocardial infarction	6 (3%)	6 (6.9%)	0 (0%)	0.006
Cardiac tamponade	0 (0%)	0 (0%)	0 (0%)	—
Cerebral infarction	1 (0.5%)	1 (1.1%)	0 (0%)	0.437

Data presented as frequency and percentage, mean ± standard deviation, or median and interquartile range. A *p* value<0.05 indicates statistical significance. Abbreviations: CN, calcified nodule.

**Table 3:** Angiographic findings and intravascular ultrasound findings compared between the patients with and without calcified nodule.

	All patients (*N* = 200)	CN (*n* = 87)	Non-CN (*n* = 113)	*p*
Angiographic findings				
Preintervention				
Lesion length (mm)	37.36 ± 17.09	35.49 ± 17.23	38.76 ± 16.93	0.196
Reference lumen diameter (mm)	2.61 ± 0.58	2.65 ± 0.64	2.58 ± 0.53	0.388
Minimum lumen diameter (mm)	0.48 ± 0.33	0.50 ± 0.37	0.47 ± 0.30	0.473
Diameter stenosis (%)	81.35 ± 12.56	81.13 ± 12.51	81.52 ± 12.66	0.830
Postintervention				
Reference lumen diameter (mm)	2.73 ± 0.45	2.78 ± 0.45	2.69 ± 0.45	0.144
Minimum lumen diameter (mm)	2.43 ± 0.40	2.46 ± 0.42	2.41 ± 0.38	0.407
Diameter stenosis (%)	10.68 ± 7.21	11.52 ± 7.83	10.04 ± 6.67	0.152
Acute lumen gain (mm)	1.95 ± 0.45	1.95 ± 0.42	1.94 ± 0.47	0.843

IVUS parameter^*∗*^	*n* = 125	*n* = 54	*n* = 71	
Mean reference vessel	3.28 ± 0.70	3.35 ± 0.74	3.24 ± 0.67	0.281
Minimal stent area	6.56 ± 2.35	6.67 ± 2.58	6.48 ± 2.17	0.648
Minimal stent area ≤5 mm^2^	32 (25.6%)	14 (25.9%)	18 (25.4%)	0.942
Minimal stent area ≤5.5 mm^2^	43 (34.4%)	19 (35.2%)	24 (33.8%)	0.872
Asymmetrical stent expansion	6 (4.8%)	5 (9.3%)	1 (1.4%)	0.084

IVUS findings: plaque characteristics				
Superficial calcification^*∗∗*^	196 (98.0%)	87 (100.0%)	109 (96.5%)	0.134
Concentric calcification	182 (91.0%)	80 (92.0%)	102 (90.3%)	0.679
Napkin ring sign	100 (50.0%)	43 (49.4%)	57 (50.4%)	0.887
Reverberation	140 (70.0%)	53 (60.9%)	87 (77.0%)	0.014
Calcium fracture	164 (82.0%)	75 (86.2%)	89 (78.8%)	0.174

Data presented as frequency and percentage or mean ± standard deviation. A *p* value<0.05 indicates statistical significance. ^*∗*^IVUS data OptiCross™ that transferred to storage source can be reviewed, but it cannot be offline analysis for measurement information. ^*∗∗*^At the other site of the calcified nodule. CN, calcified nodule; IVUS, intravascular ultrasound.

**Table 4:** Wire bias based on IVUS data and DoCE in the patients with eccentric CN/NC.

Case	Description	Maximum burr size	DoCE	Image
1	Wire bias toward CN	1.5	N	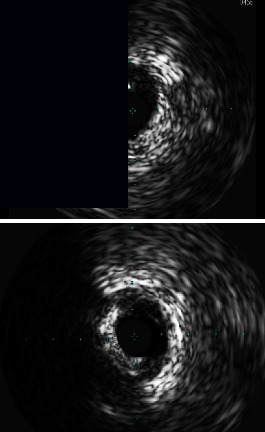

2	Wire bias toward CN	1.75	N	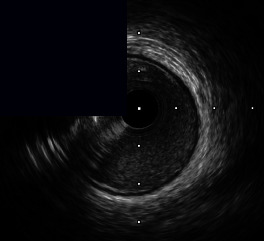

3	Wire bias opposite to CN angiographic short segment at target lesion	1.5	N	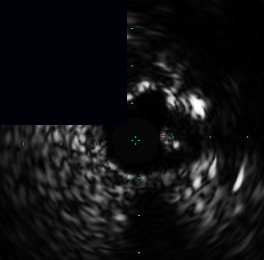

4	Wire bias opposite to CN angiographic 90% stenosis lesion at target segment	1.5	N	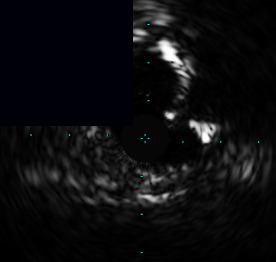

5	Wire bias opposite to CN	1.5	Y	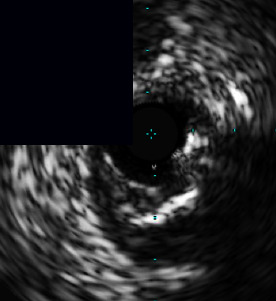

6	Wire bias opposite to CN	1.5	Y	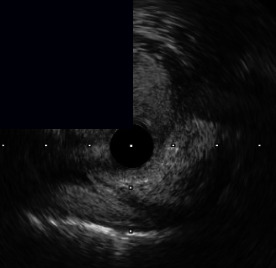

7	Wire bias opposite to CN	1.75	Y	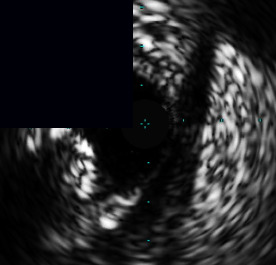

## Data Availability

The datasets used and/or analyzed in this study are available from the corresponding author on reasonable request. Identifying/confidential patient data should not be shared.
